# Influence of genetic variants of the vitamin D receptor on clinical profile in cirrhosis and hepatocellular carcinoma

**DOI:** 10.3332/ecancer.2025.1990

**Published:** 2025-09-16

**Authors:** Simone P S Lima, Rafael Fernandes-Ferreira, Beatriz J Brait, Franciana L Aguiar, Marcela A S Pinhel, Abner dos Santos Abreu, Renato F Silva, Rita C M A Silva, Doroteia R S Souza

**Affiliations:** 1 Medical School of São José do Rio Preto (FAMERP), São José do Rio Preto 15090-000, Brazil; 2 Health Science Institute, Paulista University, São Paulo 04026-002, Brazil; 3 Department of Internal Medicine, Ribeirão Preto Medical School, University of São Paulo (USP), Ribeirão Preto 14.040-904, Brazil; a https://orcid.org/0000-0001-5250-6212; * Contributed equally as first authors to this study

**Keywords:** polymorphisms, liver, VDR, hepatocellular carcinoma, vitamin D, 25(OH)-D

## Abstract

Cirrhosis is the fourteenth leading cause of death globally and significantly increases the risk of hepatocellular carcinoma (HCC). Polymorphisms in the vitamin D receptor (VDR) can influence inflammation, fibrosis progression and cancer susceptibility. We analysed the association of genetic polymorphisms of the VDR (VDR-rs2228570, VDR-rs731236 and VDR-rs7975232) in cirrhosis with or without HCC, considering clinical, biochemical profiles and survival. A total of 158 patients with cirrhosis, with or without HCC, were studied and distributed into Group 1 (G1 = 60): cirrhosis and HCC; Group 2 (G2 = 98): isolated cirrhosis and control group (G3 = 225): without liver disease. Genetic polymorphisms were analysed by real-time polymerase chain reaction; clinical and biochemical profiles were obtained from medical records. A significance level of α = 5% was adopted. The homozygous mutant for VDR-rs731236 and rs7975232 predominated in G1 compared to other groups (*p *< 0.05). For VDR-rs2228570, the homozygous mutant predominated in patients, while heterozygotes were found in controls (*p *> 0.05). A positive correlation between vitamin D and parathyroid hormone was observed in patients (*R*² = 0.3273). VDR-rs2228570 emerged as a protective factor for G2 (*p *= 0.0057) and was associated with increased survival, as was rs7975232. In conclusion, VDR-rs731236 and VDR-rs7975232 are associated with cirrhosis and HCC, with VDR-rs7975232 identified as independent predictors for isolated cirrhosis. VDR-rs2228570 confers protection and is associated with increased survival in cirrhosis, as well as a better clinical profile for both conditions in the Brazilian cohort. These findings highlight the potential clinical relevance of VDR polymorphisms as biomarkers for risk assessment and prognosis in cirrhosis and HCC.

## Introduction

Cirrhosis, a chronic liver disease, is characterised by a diffuse fibrotic process with alteration of the lobular architecture. It is classified as the fourteenth leading cause of death worldwide and the tenth in developed countries. The increase in cirrhosis, combined with risk factors (lifestyle habits and comorbidities), has led to a rising incidence of liver cancer, which currently represents the second most common cause of cancer-related deaths globally. Among primary liver tumours, hepatocellular carcinoma (HCC) stands out as the most aggressive and frequent, accounting for 85%–90% of primary liver neoplasms [1–3].

Vitamin D has been the focus of extensive research, particularly regarding its role in various cancers, including HCC [4, 5]. As a member of the secosteroid hormone superfamily, vitamin D consists of fat-soluble compounds derived from plant and animal sources (D2/ergocalciferol and D3/cholecalciferol, respectively), both of which demonstrate equivalent biological activity. Pre-vitamin D3 is located in the deeper layers of the skin, where it undergoes isomerisation through the action of UVB rays. In this process, pre-vitamin D enters the bloodstream and is transported to the liver by the vitamin D-binding protein [4, 6]. In the liver, it binds to the vitamin D receptor (VDR), initiating its first hydroxylation, which produces cholecalciferol [25(OH)D^2^ and/or 25(OH)D^3^]. It is then transported to tissues containing 1α-hydroxylase (CYP27B1). The kidneys have the highest concentration of this enzyme, leading to the formation of the active metabolite, calcitriol [1α25-(OH)_2_-D^3^] [4,6].

Recognised for its role in calcium homeostasis [6], vitamin D also possesses potential inhibitory effects on angiogenesis and inflammatory processes [4, 6]. In this context, it acts by inhibiting the activation and proliferation of T cells, as well as the suppression of pro-inflammatory cytokines [5, 8]. Furthermore, calcitriol may inhibit the growth of malignant cells by regulating the cell cycle [9] through the modulation of tumour suppressor genes [10]. Genetic alterations related to the endocrine metabolism of vitamin D may influence its synthesis and, consequently, the biological response [5].

In this context, the VDR, which belongs to the superfamily of nuclear receptors that encompasses various ligand-activated transcription factors, is particularly noteworthy. It functions as a cytoplasmic transcription factor and, upon activation by vitamin D, translocates to the nucleus, where it interacts with promoter regions of DNA to which it has affinity. This interaction results in multiple biological effects, including its role as an immunomodulator and in the regulation of inflammation [5]. This process is facilitated by the retinoid X receptor (RXR), which enhances the affinity of the VDR-DNA complex. Consequently, VDR plays a crucial role in modulating essential events for the activation of target genes [5, 11]. It is critical to underscore that approximately three thousand genes are regulated by VDR activity in the human genome, a process that is acknowledged as a genomic pathway modulated by hormonal control [12].

The single-nucleotide polymorphisms (SNPs) of VDR rs2228570, rs731236 and rs7975232, may substantially influence variations in bone mass and cancer susceptibility. The expression of VDR variants has the potential to promote inflammation and the progression of fibrosis, as well as to underscore their role in the development of HCC [13]. Therefore, mutations in the VDR gene compromise the function of the heterodimer complex [RXR-VDR-1,25(OH)₂D₃], which may lead to vitamin D resistance and alterations in the clinical phenotype [14].

Thus, this study aimed to evaluate the association of genetic polymorphisms of the VDR (VDR-rs2228570, VDR-rs731236 and VDR-rs7975232) with clinical, biochemical profiles and survival outcomes in cirrhosis, with or without HCC patients.

## Patients and methods

### Patients and ethics statements

A total of 383 individuals were studied, including 158 patients (Total Group = TG) with cirrhosis, with or without a diagnosis of HCC, under follow-up at the Liver Transplant Service and Gastrointestinal Cancer Institute of the Hospital de Base/Faculdade de Medicina de São José do Rio Preto (HB/FAMERP). Patients were selected based on a cirrhosis diagnosis established by the team’s protocol and were divided into: Group 1 (G1) – 60 patients with cirrhosis and HCC (ages 46 to 81 years) and Group 2 (G2) – 98 patients with isolated cirrhosis (ages 16 to 71 years). The control group (G3) comprised 225 individuals (ages 20 to 84 years) attending the Hemocentro – HB/FAMERP or outpatient clinics with no clinical or laboratory evidence (serum levels of aspartate aminotransferase – AST and alanine aminotransferase – ALT) of liver disease. All participants signed a Free and Informed Consent Term. The study was approved by the Research Ethics Committee of the Faculdade de Medicina de São José do Rio Preto (CEP/FAMERP; Process numbers 435/2011 and 6910/2011).

### Genotyping

All individuals underwent the collection of a 20 mL peripheral blood sample for genomic DNA extraction. Serum assays for vitamin D and parathyroid hormone (PTH), were conducted at the Central Laboratory HB/FAMERP on a subgroup of patients and controls (GTd = 30 patients from the total group: G1d = 4 with cirrhosis and HCC; G2d = 26 with isolated cirrhosis; G3d = 20 individuals without liver diseases). Following the extraction of genomic DNA, the analysis of the polymorphisms VDR-rs2228570 (C_12060045_20), VDR-rs731236 (C_2404008_10) and VDR-rs7975232 (C_28977635_10) was performed using real-time polymerase chain reaction (PCR). The final reaction volume was 5 μL, comprising 1.375 μL of DEPC-treated water, 2.5 μL of Universal Master Mix (Thermo Scientific™), 0.125 μL of TaqMan^®^ SNP Genotyping Assays probe for each polymorphism and 1 μL of diluted DNA. The reactions were conducted under the following thermal cycling conditions: 95°C for 10 minutes, followed by 47 cycles at 92°C for 15 seconds each and 60°C for 1 minute (StepOne Plus Real-Time PCR System, Applied Biosystems). Allele determination was carried out using StepOne Software v2.3 (Applied Biosystems).

### Statistical analysis

Fisher's exact test or the chi-square test was utilised to evaluate qualitative variables, including genotypic and allelic frequencies, while *t*-tests or Mann–Whitney tests were applied for quantitative variables (GraphPad InStat 3). Event-free survival curves (Kaplan–Meier) were constructed, considering the presence of at least one risk allele (StatsDirect Statistical Analysis), in conjunction with logistic regression analysis. The logistic regression model was adjusted for the following variables: age, sex, presence of viral hepatitis (B and/or C), gamma-glutamyl transferase (GGT) levels, altered serum creatinine levels, diabetes mellitus, arterial hypertension and BMI . The logistic regression equation was: logit(Y) = -6.841805 + 0.979429 (Viral hepatitis) + 1.533913 (GGT) + 0.823873* (Creatinine) + 0.074988*(Age)**. Hardy–Weinberg equilibrium (HWE) for each SNP and linkage disequilibrium (LD) were assessed using Arlequin v. 3.11 and differences in haplotypic frequencies were analysed using GraphPad Prism (version 5.0). A significance level of *p* < 0.05 was established.

## Results

### Biochemical profile

Elevated serum levels of AST were notably observed in men within G1 (65.0 U/L) compared to G2 (52.0 U/L; *p* = 0.0159; Table 1). A similar pattern was observed for GGT [G1=115 U/L; G2=84 U/L; *p* = 0.0071]. Regarding alpha fetoprotein, elevated values were predominant in G1 (men and women: 13,350 and 1,416.0 ng/mL, respectively) compared to G2 (2,805 ng/mL; *p* < 0.0001 and 4,250 ng/mL; *p* = 0.0227, respectively). No significant differences were identified between the patient groups for ALT, bilirubin and albumin levels (*p* > 0.05 for all).

The groups exhibited comparable serum levels of vitamin D and PTH. Altered values for vitamin D (<30 ng/mL) were noted in 30% of GTd and 35% of G3d, while abnormal PTH levels (<15 or >65 ng/mL) were present in 16.7% and 15% of patients and controls, respectively (*p* > 0.05). A moderate positive correlation between vitamin D and PTH (*R*² = 0.3273) was observed in GT, which was not apparent in controls (*R*² = 0.0106).

### Genetical profile and haplotypic frequencies

The homozygous wild-type genotype for VDR-rs731236 (TT) and VDR-rs7975232 (CC) was predominant in G1 (38.3% and 42.4%, respectively), whereas the heterozygous genotype was prevalent in G2 and G3 (TC = 51% and 46.7%; CA = 45.9% and 52%, respectively), with no significant differences observed between the groups (*p* > 0.05). In contrast, the homozygous mutant genotype (CC and AA, respectively) was more frequent in G1 (notably in males at 26% and females at 40%) compared to G2 (6.8%; *p* = 0.0040) and G3 (12.2%; *p =* 0.0397). For VDR-rs2228570, the homozygous mutant genotype (CC) was prominently represented in both patient groups (G1 = 48.4%; G2 = 41.8%), while the heterozygous genotype (TC) was observed in controls (45.8%; *p* > 0.05). HWE was established for all SNPs in both patients and controls (*p* > 0.05).

The LD analysis, which assesses the non-random association of alleles, revealed significant associations for rs-7975232-rs731236 in both patients and controls (*p* = 0.00001 for both), as well as for rs-7975232-rs2228570 in patients (*p* = 0.01466). Haplotype reconstruction for the VDR SNP set identified the TCC haplotype in patients (20.3% versus controls = 15.1%; *p* = 0.058) and the TAC haplotype in controls (26.2% versus patients = 20.9%; *p* = 0.0975), with a lower frequency of the TCT haplotype in both patients (5.7%) and controls (5.8%; *p* = 0.808).

### Genotypes and its association with clinical profile and survival

The comparative analysis of VDR polymorphisms, considering the presence of the polymorphic allele and the homozygous wild-type genotype, revealed a similar genotypic distribution between reduced levels of vitamin D (<30 ng/mL) and recommended levels (≥30 ng/mL) in patients and controls (*p* > 0.05). The relationship between severity criteria and genetic polymorphisms indicated that, in G1, a score of ≤20 was associated with a higher prevalence of genotypes containing at least one polymorphic allele for VDR-rs2228570 (_/C) compared to scores >20 (82.3% and 17.7%; *p* = 0.0452). In G2, a similar association was observed for scores <16 (92.9% versus ≥16 = 7.14; *p* = 0.0297). The VDR-rs2228570 polymorphism emerged as a protective factor in G2 (*p* = 0.0057).

**Figure 1.**
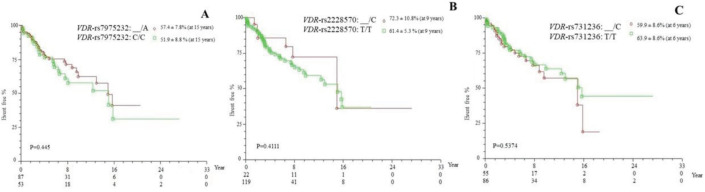
Kaplan–Meier actuarial curve for event-free survival (death), considering the polymorphisms (a) VDR-rs795232, (b) VDR-rs2228570 and (c) VDR-rs731236 in patients with cirrhosis with or without HCC, carriers of genotypes with at least one polymorphic allele (_/A, _/C and _/C, respectively), compared to the wild-type homozygous genotype (C/C, T/T nad T/T, respectively). VDR = vitamin D receptor.

The polymorphisms VDR-rs7975232 and VDR-rs2228570 were associated with increased survival in the presence of the polymorphic allele, whereas for VDR-rs731236, increased survival was observed for the wild-type genotype (TT), although without significant difference (*p* > 0.05; Figure 1).

## Discussion

Understanding the association of the molecular mechanisms involved in the progression of cirrhosis to HCC and the protective effects of VDR polymorphisms is essential for therapeutic advancements and improved prognosis for patients with this neoplasm. In this context, the present study makes a novel contribution to the literature. The polymorphisms VDR-rs7975232 and VDR-rs731236 were highlighted in this study. Mutant homozygosity (CC) for VDR-rs731236 predominated in the cohort with HCC compared to isolated cirrhosis, consistent with findings in Chinese populations [15]. Conversely, the study by Falleti *et al* [15] reported a prevalence of wild-type homozygosity (CC) among patients with the VDR-7975232 genetic variant, whereas our cohort revealed that the mutant homozygote (AA) was predominant in cirrhosis and HCC patients.

In this context, the presence of the polymorphic allele emerged as an independent factor for cirrhosis associated with HCC. VDR polymorphisms may hinder the interaction between active vitamin D and the VDR, leading to inefficiencies in this molecular complex and, consequently, impairing the transcription of genes involved in the vitamin D signaling pathway. These disruptions can compromise the regulation of the immune system, apoptosis and angiogenesis [7]. Thus, it is plausible to associate these genetic variants, which are prevalent in patients with cirrhosis and HCC, with disease progression.

The association between VDR polymorphisms and chronic liver disease has been the subject of investigation [5]. The VDR-rs731236 and rs7975232 variants, arise from single-nucleotide substitutions that do not alter the VDR protein but are implicated in the regulation of messenger RNA stability, potentially influencing gene expression, as demonstrated in animal models [5, 16]. In contrast, the mutation in VDR-rs2228570 results in a truncated protein due to a modification of the start codon [16]. In the present study, while VDR-2228570 exhibited a similar allelic and genotypic distribution between patients and controls, in accordance with the findings of Triantos *et al* [5], the presence of the polymorphic allele (_/C) conferred a protective effect against cirrhosis and was associated with an increase in survival [5]. Previous studies have indicated the predominance of the wild-type allele (T) in cases of hepatitis C and HCC within the Chinese population [17].

The HWE was assessed and confirmed for the specified SNPs in patients and controls, consistent with the literature [18], suggesting an absence of mutation and selection events, with random mating and gene flow not altering the allelic composition of the population [19]. The reconstruction of haplotypes (a combination of alleles typically inherited as a unit) demonstrated similar haplotype frequencies between patients and controls, indicating no association with risk or protection against the disease. However, Falleti *et al* [15] identified an association between VDR haplotype recombination and the disease, including VDR-rs1544410, which was not investigated in this study, in conjunction with VDR-rs7975232 and VDR-rs731236 [15].

Additionally, the recombination rate was measured, evaluating the deviation of haplotype frequency relative to the corresponding alleles in the population, specifically the LD [20]. This was observed for VDR-rs7975232 and VDR-rs731236 in both groups, corroborating Hung *et al* [21]. A similar observation was noted for VDR-rs7975232 and VDR-rs2228570, but only in patients, which is inconsistent with other studies [21, 22]. It is important to note that LD may arise from selection, mutation, genetic drift or population subdivision [23]. Therefore, further studies are necessary within the Brazilian context, considering the recognised mixed ancestry of the population [24].

Regarding the biochemical profile, elevated serum levels of GGT, ALT and AST may reflect hepatic injury, hepatic steatosis and/or oxidative stress, serving as predictors of disease and mortality [25]. In this study, as well as in other case series [26], elevated levels of AST, particularly in cirrhosis with HCC, confirmed a poorer prognosis, consistent with findings for various types of cancer [27]. Similar findings were observed for GGT, which presented as an independent factor for the disease, supporting another study highlighting GGT production by tumour cells, leading to increased serum levels [28, 29].

Moreover, there is an established inverse correlation between serum vitamin D levels and the progression of liver diseases and mortality [30–33]. However, this study observed an increase in vitamin D levels among patients. Serum levels of PTH may serve as key mediators for the induction of active vitamin D [34, 35] and should be analysed in conjunction with vitamin D levels. A negative correlation between PTH and vitamin D levels is well-documented, considering the pathway involving PTH in the influx of calcium and phosphate, which enhances vitamin D synthesis [35]. In contrast, this study found a moderate positive correlation between PTH and 25(OH)D levels exclusively within the patient group, corroborating findings from Stein *et al* [35] in a multiple sclerosis cohort. This observation suggests the potential influence of additional regulatory factors.

It is noteworthy that physiological alterations or the development of a disease state may influence the expression of the VDR in specific tissues [35]. Research in mice suggests positive regulation between VDR and PTH, involving genome editing [36]. It is well recognised that the absence of 1,25(OH)D triggers the binding of VDR to target genes, functioning as a repressor and forming complexes with histone deacetylases (HDACs), which consequently leads to gene silencing. Conversely, the presence of vitamin D promotes the release of HDAC, resulting in the recruitment of histone acetyltransferases and chromatin remodeling complexes, thereby initiating transcription [37]. Histone deacetylation contributes to chromatin condensation, resulting in VDR silencing [37] and, consequently, vitamin D resistance [14].

Thus, the positive correlation between vitamin D and PTH observed in patients may be related to dysfunctional VDR, potentially involving polymorphisms such as VDR-rs731236 and rs7975232 that could hinder the genomic pathway of vitamin D. This situation underscores the necessity of stimulating activation pathways for 25(OH)D^3^, alongside increased PTH, which stimulates renal hydroxylation enzymes [35, 38] to address the false deficiency of vitamin D. The underutilisation of vitamin D may reflect a clinical phenotype of vitamin D resistance, warranting further investigation in larger cohorts and diverse populations, as well as additional biochemical parameters.

There was also a similarity in the genetic profile concerning disease severity. However, another case series reported an association between VDR polymorphisms and more severe scores [39]. It is conceivable that patients with cirrhosis and higher severity scores may succumb to their condition before progressing to HCC, which could explain the similarities between the groups. According to the Model for End-Stage Liver Disease criteria, which assesses 3-month mortality risk, this study found that the presence of the polymorphic allele for VDR-rs2228570 (_/C) was associated with lower scores and increased survival. This mutation results in a truncated protein; however, it is possible that the genomic function of VDR remains intact [5] or that the presence of the polymorphic allele may confer protection against cirrhosis, as evidenced in this cohort.

In conclusion, VDR-rs731236 and VDR-rs7975232 are associated with cirrhosis and HCC. Furthermore, VDR-rs2228570 appears to confer protection and improve survival in cirrhosis, as well as enhance the clinical profile for cirrhosis and HCC within the Brazilian cohort. Further research with larger cohorts is necessary to validate these associations and to explore the molecular mechanisms through which VDR polymorphisms influence the progression of cirrhosis and the development of HCC, as well as their impact on clinical outcomes and treatment response.

## Conflicts of interest

The authors of the article titled ‘Influence of Genetic Variants of the VDR on Clinical Profile in Cirrhosis and Hepatocellular Carcinoma’, submitted to *ecancermedicalscience*, declare that we have no conflicts of interest related to the research, funding or any other aspect of the study presented.

## Author contributions

Simone PS Lima: Conceptualisation; Data curation; Methodology; Roles/Writing - original draft; Writing - review; editing. Rafael Fernandes-Ferreira: Formal analysis; Funding acquisition; Investigation; Methodology; Writing - original draft and review. Beatriz J Brait: Data curation; Methodology. Franciana L Aguiar: Data curation; Methodology. Marcela AS Pinhel: Formal analysis; Investigation; Methodology; Writing - original draft. Abner S Abreu: Formal analysis; Investigation; Methodology; Writing - original draft and review. Renato F Silva: Conceptualisation; manuscript review. Rita CMA Silva: Conceptualisation; Funding acquisition; Roles/Writing - original draft. Doroteia RS Souza: Conceptualisation, Project administration; Funding acquisition; Resources Writing - original draft; Writing - review; Editing; Supervision.

## Figures and Tables

**Table 1.** Risk profile by logistic regression analysis in the total patient group (TG) = cirrhosis with or without HCC, in patients with cirrhosis and HCC (G1) and in patients with isolated cirrhosis (G2).

	TG			G1			G2			G1 x G2		
Covariate	Odds ratio	Confidence interval 95%	*p	Odds ratio	Confidence interval 95%	*p	Odds ratio	Confidence interval 95%	*p	Odds ratio	Confidence interval 95%	*p
Smoking	2.42	1.22–4.81	**0.0112**	-	-	**-**	2.86	1.08–7.59	**0.0344**	-	-	-
Alcohol	19.69	9.95–38.95	**<0.0001**	19.87	4.61–85.64	**<0.0001**	17.58	6.41–48.17	**<0.0001**	-	-	-
DM	11.22	3.87–32.58	**<0.0001**	17.90	3.40–94.27	**0.0007**	14.24	3.23–62.86	**0.0005**	-	-	-
AST	-	-	**-**	9,923.55	6.02–16,361,964.68	**0.0149**	40,006.24	1.63–979,777,009.06	**0.0399**	-	-	-
ALT	2,202.76	9.55–507,785.13	**0.0056**	-	-	-	-	-	-	-	-	-
VDR-rs7975232 _/A	-	-	-	-	-	**-**	3.76	1.00–14.15	**0.0500**	-	-	-
VDR rs2228570 _/C	-	-	-	-	-	**-**	0.19	0.06–0.62	**0.0057**	-	-	-
Age	1.04	1.01–1.07	**0.0062**	1.09	1.03–1.16	**0.0033**	-	-	-	1.08	1.04–1.21	**0.0002**
Viral hepatitis										2.66	1.22–5.83	**0.0144**
GGT										4.64	1.88–11.44	**0.0009**
Creatinine										2.28	1.04–4.98	**0.039**

*Chi-Square Test or Fisher; DM = Diabetes mellitus; AST = Aspartate aminotransferase; ALT = Alanine aminotransferase; VDR = Vitamin D receptor; GGT = Gamma-glutamyl transferase; Blank Space = Analysis not performed; = *p*-value > 0.05

**Table 2.** Demographic profile, lifestyle habits and comorbidities in the total patient group (TG), in patients with cirrhosis and HCC (G1), with isolated cirrhosis (G2) and in individuals without the disease (G3).

Variables	GT (*N* = 158)		G1 (*N* = 60)		G2 (*N* = 98)		G3 (*N* = 225)		*Valor-*p*			
									GT x G3	G1 x G2	G1 x G3	G2 x G3
Sex	***N***	**%**	***N***	**%**	***N***	**%**	***N***	**%**				
Male	124	78,5	50	83.3	74	75.5	127	56.4	**<0.001**	**0.0002**	**0.0018**	0.3361
Female	34	21.5	10	16.7	24	24.5	98	43.6				
Lifestyle habits	***N***	**%**	***N***	**%**	***N***	**%**	***N***	**%**				
Smoking	74	46.8	27	45.0	47	48.0	45	20.0	**<0.0001**	0.8434	**<0.0001**	**<0.0001**
Alcohol	98	62.0	39	65.0	59	60.2	26	11.6	**<0.0001**	0.6643	**<0.0001**	**<0.0001**
Comorbidities	***N***	**%**	***N***	**%**	***N***	**%**	***N***	**%**				
DM	41	26.0	16	26.7	25	25.5	8	3.6	**<0.0001**	0.8721	**<0.0001**	**<0.0001**
SAH	53	33.5	18	30.0	35	35.7	70	31.1	0.6959	0.5723	0.9934	0.4947

*p* = Significance level <0.05; *Chi-square or Fisher's test; N = Number of individuals; DM = Diabetes mellitus; HAS = Systemic arterial hypertension

## Supplementary Material

### Inclusion and exclusion criteria

Inclusion criteria were established for patients with cirrhosis, regardless of etiology, with or without hepatocellular carcinoma (HCC). Exclusion criteria encompassed regular use of vitamin D-containing supplements, conditions associated with decreased serum levels of vitamin D and vitamin D receptor polymorphisms, including type 1 diabetes, Crohn's disease and Graves' disease [1], Hansen's disease [2], autoimmune hepatitis [3], psoriasis [4] and multiple sclerosis [5].

The diagnosis of cirrhosis was performed in accordance with a pre-established protocol set forth by the respective services, encompassing clinical aspects (telangiectasias, palmar erythema, gynecomastia, jaundice, hepatosplenomegaly, ascites, collateral circulation and edema of the lower extremities), laboratory findings (hypoalbuminemia, prolonged coagulation profile and thrombocytopenia) and imaging studies (ultrasound and/or computed tomography and/or magnetic resonance imaging indicating an irregular, nodular and heterogeneous liver, signs of portal hypertension, as well as upper gastrointestinal endoscopy revealing esophageal varices and/or congestive gastropathy) and/or biopsy [6]. For the diagnosis of HCC, the guidelines established by the American Association for the Study of Liver Diseases were considered [7]. Patients were staged in accordance with the Child-Pugh classification [8], which evaluates the severity of cirrhosis and is employed to determine eligibility for transplantation. Additionally, the Model for End-Stage Liver Disease (MELD) score was utilised to assess the risk of mortality within 3 months and to guide patient allocation on the transplantation list [9]. A questionnaire, along with both electronic and physical medical records, was employed to obtain data regarding comorbidities (hypertension and diabetes mellitus (DM)) and lifestyle habits (smoking and alcohol consumption). Furthermore, the assessment of patients encompassed serum levels of AST, ALT, gamma-glutamyl transferase and alpha-fetoprotein.

### Statistical analysis

The DIMAM 1.0 software was employed for the estimation of sample size.

### Results

#### Demographic, lifestyle habits and clinical profile

A predominance of the male sex was observed, particularly in G1 compared to G2 and G3 (*p* = 0.0002 and *p* = 0.0018, respectively). The prevalence of smoking, alcohol consumption and DM was significantly higher among patients compared to controls (*p* < 0.0001; Table 2). Regarding the clinical profile, viral hepatitis B and C were noted with prevalences of 52.6% in G1 (G2 = 43.4%; *p* > 0.05). Additionally, cirrhosis due to other etiologies (cryptogenic, *Budd-Chiari* syndrome, ductopenic syndrome, hemochromatosis, biliary cirrhosis, sclerosing cholangitis or portal fibrosis) was prevalent in G2 (13.9%; G1 = 4.1%; *p* = 0.0266).

Concerning disease severity, as classified according to the Child-Pugh system, type A predominated in both groups (G1 = 52.5%; G2 = 52%; *p* > 0.05). For the MELD criteria, a predominance of scores ≤20 was noted in G1 (91.4%; G2 = 23.5%; *p* = 0.0294). Furthermore, mortality was significantly higher in G1 (56.7%; G2 = 24.5%; *p* < 0.0001).

### Discussion

#### Demographic and lifestyle habits

There was a prevalence of male sex and an increase in age, particularly among patients with cirrhosis and HCC, corroborating findings from other studies [10, 11]. Furthermore, alcohol consumption, smoking and DM were observed in both patient groups [12]. The presence of alcohol consumption was associated with a higher likelihood of cirrhosis, with or without HCC [13], as well as smoking and DM [14].

Alcohol intake causes damage to hepatic tissue due to the action of endotoxins, as well as oxidative stress and inflammation, leading to fibrosis in liver tissue, which contributes to the development of cirrhosis and HCC [15]. Additionally, tobacco contains over 40 active compounds metabolised by the liver, some of which have already been established as contributors to carcinogenesis [11, 16]. In this context, there is an induction of the immune system with the production of antibodies, helper T cells and cytotoxic T cells, along with an increase in inflammatory cytokines [16], which can damage hepatocytes, resulting in necrosis and inflammation [17] and consequently, malignant tumourigenesis [18].

As a risk factor for cirrhosis and HCC, DM was prevalent among the patients, corroborating other studies [19, 20]. The crucial role of the liver in glucose metabolism underscores the relationship between DM and chronic liver diseases, such as hepatitis, steatosis and cirrhosis. Furthermore, liver cell damage induced by type 2 DM involves insulin resistance and hyperinsulinemia, as well as inflammation, cellular proliferation, inhibition of apoptosis and mutations in tumour suppressor genes [20].
